# Parental brain: cerebral areas activated by infant cries and faces. A comparison between different populations of parents and not

**DOI:** 10.3389/fpsyg.2015.01625

**Published:** 2015-10-21

**Authors:** Giulia Piallini, Francesca De Palo, Alessandra Simonelli

**Affiliations:** Department of Developmental Psychology and Socialization, University of PaduaPadua, Italy

**Keywords:** parents, adults, parental brain, infant cries, infant faces

## Abstract

Literature about *parenting* traditionally focused on caring behaviors and parental representations. Nowadays, an innovative line of research, interested in evaluating the neural areas and hormones implicated in the nurturing and caregiving responses, has developed. The only way to permit a newborn to survive and grow up is to respond to his needs and in order to succeed it is necessary, first of all, that the adults around him understand what his needs are. That is why adults’ capacity of taking care of infants cannot disregard from some biological mechanisms, which allow them to be more responsive to the progeny and to infants in general. Many researches have proved that exist specific neural basis activating in response to infant evolutionary stimuli, such as infant cries and infant emotional facial expression. There is a sort of innate predisposition in human adults to respond to infants’ signals, in order to satisfy their need and allow them to survive and become young adults capable of taking care of themselves. This article focuses on research that has investigated, in the last decade, the neural circuits underlying parental behavioral responses. Moreover, the paper compares the results of those studies that investigated the neural responses to infant stimuli under different conditions: familiar versus unknown children, parents versus non-parents and normative versus clinical samples (depression, addiction, adolescence, and PTSD).

## Introduction

Parents play an essential role in the survival and development of the infant and the dyadic relation between parents-infants represent the first, and most important, interaction for the baby. It structures underlying neural mechanisms responsible of its typical or atypical development. Adult–infant relationships has a long evolutionary history, which suggests that specific brain circuits might mediate adult responsiveness to infants (Caria et al., 2012; Esposito et al., 2013). The efficacy of such relationship depends, among other things, on certain children’s characteristics, such as facial expressions morphology and communicative signals (e.g., cry, laugh, gaze, gestures) that activates appropriate caregiving behaviors in the adults (Bornstein, 2002; Bornstein et al., 2008; Doi and Shinohara, 2012; Esposito et al., 2013). By the way, newborn infants communicate their needs and physiological states mainly through crying and facial expression. Therefore, crying is the principal resource the immature newborn possesses to arouse parental care, especially from a distance or when infants are out of their sight (Soltis, 2004; Konner, 2010); at the same time infant facial expression represents an important resource of non-verbal communication between parents and their infants. There is a sort of mutual regulation between partners: at the infant signal, the adult is supposed to respond adequately. Such reciprocal adjustment suggests there might be a biological predisposition in establishing shared relations aimed at offspring protection and care, which has to be provided by an adult able to interpret and respond to the infant’s needs.

Being a parent and adequately respond to the offspring necessities requires a huge amount of cognitive resources allowing parents to put into practice suitable caregiving behaviors and in case of parental psychopathology (e.g., depression, post-partum depression, drug addiction/dependence, etc.) these abilities may result substantially compromised, leading to inevitable consequences on the infants’ development and wellbeing, as well as on the establishment of a healthy and functional parent–infant relationship.

Still little is known about neural substrates and functional mechanisms underlying influences of adult’s ability to read infant’s cues and to respond coherently with them. An innovative line of research has employed neuroimaging techniques to identify possible modifications to neural circuitries that accompany parenthood, providing a greater level of understanding of parental processes and how psychopathology may influence parenting at a neurobiological level (Squire and Stein, 2003). This research has employed different methods to study these processes, for example functional magnetic resonance imaging (fMRI), electroencephalography (EEG) and event-related potentials (ERPs), near infrared spectroscopy (NIRS) and magneto-encephalogram (MEG) techniques can be useful to identify the neuroanatomy of parental brain, the development of neural circuits that accompany parenthood and to explain parental processes and how all those factors may be influenced by psychopathology (Swain, 2011).

The most common technique used by these studies is fMRI, a non-invasive technique, which may be used to obtain data on the cerebral basis of human parental behavior and thoughts. Measuring physiological and blood-oxygen-dependent signals in response to infant salient cues/stimuli fMRI allows collecting functional and structural data. This technique has optimal spatial resolution and it allows to se “where” the stimuli activate the neural circuitry. On the other hand, EEG technique give precise temporal resolution, affording the opportunity to explore, with millisecond accuracy, the temporal dynamics of stimulus processing, so that it helps understanding “when” the neural responses to infant affective cues take place. EEG waveforms reflect voltages produced by 1000s of synchronized postsynaptic potentials of cortical pyramidal neurons, measured at the surface of the scalp via electrodes placed according to the international 10–20 system (Jasper, 1958; Luck, 2005).

In this article we present a systematic review of the literature of the last decade about the parental neural response to infant salient stimuli (faces, cries, videos, etc.). In accordance with general aims of systematic reviews, our purpose was to provide an up-dated state of the art, which synthesizes the work in this area of knowledge. Search engines, PsycINFO, PubMed, and Google Scholar, were systematically searched using specific key words, such as “parental brain,” parenting, “brain basis,” “areas activated,” “infant cries,” “infant faces” and so on. Seventy-six papers were initially selected. Only 50 studies that fulfilled certain criteria, such as the investigation of neural and hormonal activity in response to infant cues (cries, faces, behaviors), were used in the review process.

We divided the review in sections: the first section focuses on studies which have investigated the neural basis activated in women, who were mothers, in response to infant salient stimuli (infant faces and infant cries); the second section analyzed those studies which have investigated some hormonal factors (associated to the type of delivery, breastfeeding, assumption of hormones, etc.) which may influence, in some way, the neural response to the same stimuli in mothers; the third part focuses on the investigation about gender and parental status differences in response to infant stimuli; finally, the last section discusses those studies which have investigated the fact of being a parent under clinical condition (specifically depression and addiction).

## Neural Basis of Parental Responses to Infant Cries and Faces

For infants, communicative signals include both facial and vocal components. Vocalizations, e.g., distress cries, allow the infant to capture the attention of a caregiver from a distance. The ability to respond to infant vocalizations is essential for parental responsivity (MacLean, 1990). At the same time, facial configuration of infants is thought to spontaneously attract attention and evoke caregiving in adults. Such features are viewed as pleasant and rewarding, they include large eyes and pupils, small noses and mouths, and a large forehead (Hall Sternglanz et al., 1977; Hildebrandt and Fitzgerald, 1979). There is an apparently universal and spontaneous preference for infant facial features, conserved across multiple species (Sato et al., 2012). In humans, the increased responsiveness and activity in cerebral areas implicated in communicative, infant-directed behavior is present in both parents and non-parents and specific to human infant faces, but not to faces of other infant mammals or adult faces (Caria et al., 2012). This reflects the specificity of infant-related responses, and indicates that specialized responses to infants can transcend the biological adult–infant relationship.

### Neural Response to Infant Cries

As we said, cry is the primary “strategy” the newborns have to elicit parental care (Konner, 2010). It represents the first real communicative signal infants posses in the very first social interactions, to express their own needs, to communicate with the environment and to elicit a caregiving behavior in the adult (Newman, 2007; Venuti and Esposito, 2008). It is a universal communication signal; it exists both in humans and in animal, and it provokes a universal response of approach and caring (Zeifman, 2001; Newman, 2007). The adult responsiveness to these signals is necessary to optimize the chances of survival for the individual, and for the species. The human adult brain seems to be programmed to select, elaborate and identify such type of signal and to prepare the individual for the protection and caring behavior. These mechanisms seem to supervise the individual survival and the species prosecution.

The first authors who tried to study the cerebral activity of human mothers while listening infant cries were Lorberbaum et al. (1999). Starting from Mac Lean’s thalamocingulate theory of maternal behavior in animals, they accurately anticipated that infant crying would selectively activate thalamus, cingulate, medial and prefrontal circuits in mothers while listening an audio-taped 30-s standard baby cry, not from their own infant (Lorberbaum et al., 1999).

From that moment, many studies have developed, trying to understand and to explain this mechanism. Laurent and Ablow (2012a), investigated trough fMRI the neural response of 22 primiparous mothers (*M* age = 24.1 years, *SD* = 4.1), of 15–18-month old infants, to own and unfamiliar infant’s cry and a control sound, related to infant attachment classification, evaluated trough a separate Strange Situation Procedure (SSP). Mothers were screened for psychopathology using the Structured Clinical Interview for the DSM-IV (SCID) and none of them met criteria for major depressive symptoms. Infants were classified as Avoidant, Resistant, Disorganized, or Secure based on their patterns of SSP behavior. Authors found that mothers of less secure infants maintained greater activation to their cry in left parahippocampal and amygdala regions and right posterior insula; mothers of infants showing more avoidant or contact maintaining behaviors during the SSP, displayed reduced response across left prefrontal, parietal and cerebellar areas involved in cognitive control and attentional processing; finally, mothers of infant exhibiting more disorganized behaviors showed reduced response in bilateral temporal and subcallosal areas relevant to social cognition and emotion regulation.

Some studies examined maternal sensitivity, intrusiveness and mother–infant dyadic harmony as correlates of mothers’ neural responses to the cries of their own infants (Musser et al., 2012). Twenty-two primiparous mothers were observed during an interaction with their infants at 18 months postpartum and their behavior was coded on the dimensions of sensitivity, intrusive–coercive control, and overall dyadic harmony. In a separate functional neuroimaging session, mothers were exposed to own infant’s cry sound, as well as unfamiliar infant’s cry and control sounds. Positive correlations were found between sensitive behaviors of mothers and activation in the right frontal pole and Inferior Frontal Gyrus (IFG) to their infant’s cry compared to unfamiliar cry, intrusiveness was positive correlated with activity in the left anterior insula and temporal pole while mothers who had more harmonious interactions displayed greater activation in left hippocampal regions.

These studies, which measured maternal behaviors and neural response at 15–18 months, also raises questions about temporal precedency. More studies should examine concurrent and prospective effects of maternal neural responses across early and later postnatal periods.

### Neural Response to Infant Faces

There are several studies, mostly fMRI, investigating the neural response to infant faces in mothers, because of the importance which infants’ facial expression performs in allowing the caregiver taking care of him. An infant’s happy face represent one of the most salient and rewarding stimuli for a mother for its emotional valence and its evolutionary meaning.

Studies on mothers reported grater brain response for own compared to familiar infant faces in the amygdala, anterior insula (cerebral structures associated with the emotional response), superior temporal sulcus (associated with the Theory of Mind, ToM), anterior and posterior cingulate cortex (ACC and PCC) and prefrontal regions associated with memory processes (Leibenluft et al., 2004). Other studies shown that own child’s face, compared to unknown children’s faces, activates regions involved in cognitive processes (dorsolateral prefrontal cortex -dlPFC), emotional processes [insula, medial prefrontal cortex (mPFC), and anterior cingulate cortex (ACC)] and motor processes (Strathearn et al., 2008) thalamus, temporal cortex (Barrett et al., 2012), orbitofrontal cortex (OFC) (Nitschke et al., 2004; Minagawa-Kawai et al., 2009). Recently, Esposito et al. (2015) in an EEG investigation, found that when 21 primiparous mothers (*M* age = 32.06 years, *SD* = 4.66) were exposed to their own 3- to 6-month old infants faces they shown an immediate brain response, in contrast when they look at an unfamiliar but appearance-matched infant’s face the cortical activation observed was similar but differed in magnitude in the opposite direction (Esposito et al., 2015).

Besides, Strathearn et al. (2008) in an event-related fMRI study investigated the activity of 28 primiparous women when shown face images of own 5- to 10-month old infant and a matched unknown infant. They were shown with sixty unique stimuli (own-happy, own-neutral, own-sad, unknown-happy, unknown-neutral, and unknown-sad) for 2 s each, with a variable 2- to 6-s interstimulus interval. Authors shown that only own-babies’ different emotional expressions (sad, happy, and neutral), and not others’, modulates the activation of dopaminergic circuits involved in the parental caregiving, especially the “happy” emotional expression. This finding support the idea that own-infant’s smile represents a positive reinforcement to the mother’s behavior; it represents a rewarding stimuli to the mother and reinforces her caregiving behavior. This mechanism may have a facilitating function in establishing a positive emotional circuit during the attachment relationship’s development (Strathearn et al., 2008). Similarly, Lenzi et al. (2009) recruited 16 primiparous mothers (mean 33.7 years) of infants 6- to 12-month old, without any psychopathological symptoms. Regional brain activation was assessed by measuring changes in blood-oxygen-level–dependent (BOLD) fMRI signal. Authors found stronger activation during emotional expression in the amygdala and insula in mothers observing and imitating faces of their own and others’ children, specifically they found that joy expression evoked a stronger response in right limbic and paralimbic circuits (Lenzi et al., 2009).

Thinking about the contribution of the infant’s affect to maternal brain function other study tried to investigate the maternal neural response of mothers to videos. Ranote et al. (2004) recruited ten healthy mothers with infants aged between 4 and 8 months old. Three of the mothers were primiparous; five had two children and two had three children. Ranote et al. (2004) showed to the mothers alternated blocks of videos of own and unfamiliar children and neutral videos. They found higher activity in bilateral cerebellum, visual processing regions and postcentral gyri in response to infant compared to neutral videos. Comparing own vs. unknown infants, higher activity was found in the left amygdala, right mPFC, right dlPFC, and bilateral OFC (Ranote et al., 2004).

On the other hand, Noriuchi et al. (2008) compared the response of thirteen healthy mothers (*M* age = 31.1 years, *SD* = 2.2) of children 12- to 20-month old, viewing silent videos of their own and other infants in play or separation circumstances. They found an increased activity, associated with the detection and recognition of own baby images, in cortical orbitofrontal areas (OFC), anterior insula, and precuneus, as well as subcortical regions, including the periaqueductal gray and putamen, regions operating in arousal and reward learning. Furthermore, they found strong and specific differential responses of mother’s brain to own infant’s distress in substantia nigra, caudate nucleus, thalamus, posterior and superior temporal sulcus, anterior cingulate, dorsal regions of OFC, right IFG, and dorsomedial prefrontal cortex (dmPFC; Noriuchi et al., 2008). They interpreted OFC and related activations as part of circuits required for the execution of well-learned movements. They also found correlations in OFC with own baby response and happiness as well as to their own distressed baby response in the superior temporal regions. Moreover Wan et al. (2014) included twenty healthy mothers (12 primiparous) of 4- to 10-month old healthy infants in their study and found that, viewing 30-s blocks of video of own 4–9 month infant compared to an unfamiliar matched infant, mothers shown higher activation in the precuneus, medial frontal gyri and right superior temporal gyrus (Wan et al., 2014). These results are consistent with the importance of these areas in social thoughts and behaviors.

Moses-Kolko et al. (2010), then, investigated how individual differences in mood anxiety in early post-partum are related to brain response to infant stimuli; anxiety has powerful impact on the motivation to the mother. Mothers experiencing higher levels of anxiety and parental distress and lower mood have demonstrated less amygdala responsiveness to own infant’s facial expressions (Moses-Kolko et al., 2010; Barrett et al., 2012).

Only few researches have investigated maternal neural responses to infant facial expression trough EEG and ERPs techniques. Fraedrich et al. (2010) made a first attempt to relate attachment representation with brain activity during emotional perception. They assessed 17 psychologically and neurologically healthy women (*M* age = 40.5 years, *SD* = 4.2) and found that insecure mothers, compared to secure, showed a more pronounced negativity in the face-sensitive N170 component meaning a difference in basal cortical face-processing in mothers with different attachment representation. Secure mothers seem to be more capable in face perception and thus they may be better able to detect social stimuli, to perceive infant emotional expression and to use them for social interactions (Fraedrich et al., 2010).

The picture that appears from these researches is a set of programmed structures and even circuits connected with the parental task and behaviors. This circuits incudes thalamus, insula, PFC, OFC, and ACC. These areas associated with parental response seems to overlap with circuits involved in reward and empathic processes (e.g., PFC), highlighting an intuitive close association between those tree human functions. Besides that, it has been shown that, equal circuits, mothers show different pattern of activity in terms of intensity and reactivity, when exposed to own child vs. other children (familiar and unknown); meaning non just a specie-specific response to the infant stimuli, but a more enhanced response when the infant are their own.

All the studies presented show similar limitations. The most important one is the modest size of the samples: each research included a small number of participants – from 10 to 22 (except for Strathearn et al., 2008). Larger samples of participants should be recruited to increase power and allow examination of different and more specific aspects of each research.

## Hormonal Factor which May Influence the Response of Mothers

Literature has identified brain regions related to maternal behaviors, and little research has investigated the neurobiological mechanisms underlying the relationship between maternal behavior and the hormonal changes that occur during and after pregnancy or related to that. A range of early situations surrounding the birth of a child affects postpartum hormones, parental behavior and infant wellbeing. Many studies highlights how, the internal hormonal equilibrium of the mother, can be part of the environmental maternal factors, which can positively influence the caregiving or not.

Many researches have sustained the importance of breastfeeding for supporting closer mother-infant interaction and infant socio-emotional development. Kim et al. (2011) recruited 17 mothers of healthy infants, from 2 to 4 weeks post partum, and divides them according to their feeding method (breastfeeding exclusively vs. formula-feeding exclusively) to investigate their neural activation in response to infant auditory stimuli. Breastfeeding mothers demonstrated greater activation in the superior frontal gyrus, insula, precuneus, stratum and amygdala, when listening to their own baby-cry as compared to formula-feeding mothers. This result suggests links between breastfeeding and greater response to infant cues in brain regions implicated in maternal-infant bonding and empathy during early post-partum (Kim et al., 2011). One possible mechanism underlying the relationships between breastfeeding and greater activations in the maternal circuits could be due to the effects of the oxytocin, a neurohormones involved in nurturing. Oxytocin, is synthesized in the hypothalamus and released from the posterior pituitary; it stimulates milk release at the mammary glands. It has been shown that oxytocin also facilitates other maternal behaviors in animal studies (Febo et al., 2005). Higher levels of peripheral oxytocin are associated with sensitive and synchronous parental behaviors in human mothers and fathers (Feldman et al., 2007). Such cerebral activation may facilitate greater maternal sensitivity as infants enter their social word.

A range of other circumstances around the dyad may affect the maternal capacity of perceiving and elaborate the infant stimuli. An example of such variability may be the type of delivery; Swain et al. (2008) tried to investigated whether there were any differences in the neural circuits activated in mothers responding to infant cues due to the type of delivery they had. They recruited a sample of 12 mothers (six who delivered vaginally and six who had an elective cesarean section delivery) and conducted a fMRI, 2–4 weeks after delivery. Authors found that mothers with natural childbirth showed higher neural activation in subcortical areas (hypothalamus and pons’ regions) in listening their own baby’s cry, compared to Cesarean section delivery mothers. Those regions are involved in the oxytocin neural regulation. Oxytocin is released from the uterine contractions during the delivery and during breastfeeding. Swain et al. (2008) reached to the conclusion that the higher activation in cerebral areas linked with oxytocin, in mothers with natural delivery, may be associated to events happened during the delivery itself. It can be related with a better ability to recover in the mothers with natural delivery and a higher empathic sensibility through their baby’s signals (Swain et al., 2008). One of the most important limits of this study regards the sample size, it includes only six mothers per group and the samples should be enlarged. Moreover, futures studies should examine whether the same effects are maintained several months after delivery.

Then, Laurent et al. (2011) investigated the maternal neural activity in response to infant cry, related to hypothalamic-pituitary-adrenal (HPA) axis regulation with their infants. They recruited primiparous mothers of 15–18-month-olds infants and scanned them with fMRI while listening to the cry sounds of their own, an unfamiliar infant and a control sound. Salivary cortisol was collected at four timepoints in a separated SSP session. Mothers who showed less HPA reactivity showed increased activation to the cry of their infants relative to control sound across right insula, bilateral OFC and anterior cingulate-medial prefrontal cortex (Laurent et al., 2011). On the other hand, Bos et al. (2014) showed that in non-parents males cortisol administration, compared to placebo administration, significantly increases posterior hippocampal activation selectively toward infant crying and not toward a control sound (Bos et al., 2014).

An other hormone, which has been investigated as a responsible for some differences in parental responses, is testosterone. Bos et al. (2010) measured the neural responses of sixteen young women while listening to crying infants in a double blind, placebo-controlled, counterbalanced, testosterone administration experiment. They fund heightened activation in the testosterone condition compared to placebo in the thalamocingulate region, insula and the cerebellum in response to crying, confirming him role of the thalamocingulate circuit in infant cry perception and suggesting exogenous testosterone act on the thalamocingulate circuit to upregulate parental care.

## Gender Differences in New, Expectant or Non-Parents

Becoming a parent involves a wide neuro-hormonal restructuring that prepares for the expression of adequate caregiving. Across mammalian, pregnancy and childbirth are associated with evident changes in maternal brain areas involved in motivation, nurturance and attention and, studies in bi-parental species, reveal analogous alterations in fathers’ brains, depending on the exposure to infant signals. Nowadays, only little is known about the psychological and physiological changes that expectant fathers experience before the birth of their first child or right after that, and it remains still unclear whether fathering, as mothering, involves integration of limbic and cortical circuits and is mediated by processes related to pair bonding as in other bi-parental mammals. Storey et al. (2000) measures hormone concentrations and responses to infant stimuli in expectant and new fathers, living with their couples, to determine whether men can experience variations analogous to the dramatic shift seen in pregnant women. They recruited 34 couples and took blood samples at one of four times either before or after the birth of their babies. Authors concluded that men and women had similar stage-specific differences in hormone-levels, including higher concentrations of prolactin and cortisol in the period just before the childbirth and lower postpartum concentrations of sex steroids (testosterone and estradiol). Hypothesizing that gender and experience would affect the neural responses to baby cry and laughter Seifritz et al. (2003) examined 10 women (*M* age = 31.6 years, *SD* = 4.5) and 10 men (*M* age = 36.2 years, *SD* = 4.7) with children younger than 3 years (*M* age = 1.3 years, *SD* = 0.8), and 10 women (*M* age = 27.6 years, *SD* = 3.7) and 10 men (*M* age = 28.4 years, *SD* = 4.8) without children. Using an event-related design, measuring localized brain responses to brief 6-s events, they found that over the entire sample, crying and laughing baby stimuli produced more activity in a small portion of the auditory cortex (AC), the Heshyl’s gyrus. Further, they reported that women have a decrease in activity to both baby cry and laughter in the anterior to these brief signals, which was not present in men (Seifritz et al., 2003). This is contrary to other studies (Lorberbaum et al., 1999; Lorberbaum et al., 2002; Swain et al., 2003) probably due to the different stimuli presented to the new parents in those studies. Finally, the response pattern changed fundamentally with parental experience: in the amygdala and limbic regions, parents despite of sex, showed stronger activation from crying, while non-parents showed stronger activation from laughing. These data represent the first steps into the study of gender and experience-dependent aspects of parental brain circuitry. From that point the research have developed; to investigate individual differences in tendencies to engage or withdrawal from motivationally relevant stimuli, Montoya et al. (2012) used fMRI to scan seventeen nulliparous women (*M* age = 22.7 years, *SD* = 2.9) exposing them to novel infant cries of two distress level (low and high) and unknown infants faces of varying affects (happy vs. neutral vs. sad). They found that infant cries activated bilateral superior and middle temporal gyri (STG and MTG) and precentral and postcentral gyri and the activation was grater for low- relative to high-distress cries. Happy relative to neutral faces activated the ventral striatum, caudate, ventromedial prefrontal (vmPFC) and OFC, while sand vs. neutral faces activated the precuneus, cuneus and PCC (Montoya et al., 2012).

In mixed sample of both women and men, parent and not, was shown that face processing of both adult and infant faces elicits similar waves of activity in the visual areas, from the striate cortices along dorsal and ventral pathways (Kringelbach et al., 2008); furthermore, participants of both gender showed more significant activity in the medial OFC when viewing infant faces compared to adult faces. The same pattern of activity was found when the sample was restricted to participants who were not parents (Kringelbach et al., 2008).

Similarly, Caria et al. (2012) hypothesized that non-parents’ processing of unfamiliar infant faces compared to adult faces would activate brain circuits involved in preparation for communicative and interactive responses as well as reward circuits shown to mediate attachment and caregiving behaviors in parents toward their own children. They recruited sixteen healthy adults non-parents (nine females and seven males *M* age = 28.06, *SD* = 5.66) and they scanned them with fMRI while viewing pictures of infant and adult faces. Authors fund that human infant faces activated several brain systems including lateral premotor cortex, other motor areas, cingulate cortex, anterior insula and thalamus. The same regions preferentially responded to human infant faces than to animal infant faces, suggesting species-specific adult brain responses (Caria et al., 2012). From an evolutionary perspective, adults’ grater responsiveness to human infant cues has a clear adaptive value as it supports progeny survival.

Investigating, tough, the neural activity of nine women (*M* age = 24.3 years, *SD* = 3.2) and nine men (*M* age = 27.8 years, *SD* = 6.4), without own children, in response to crying and laughing, compared to vocalization-derived control stimuli, Sander et al. (2007) found stronger activation in amygdala and ACC of women in response to natural laughing and crying, whereas the control stimuli elicited stronger activation in men. Independent of listeners’ gender, AC and PCC were more strongly activated by the control stimuli than by infant laughing or crying. The stronger activation in amygdala and ACC in women may be explained as a gender-predisposition for responding to preverbal vocalizations, while the gender-independent similarity of activation pattern in PCC and AC may reflect a more deep level of cognitive processing (Sander et al., 2007). More studies have found a coordination between mothers’ and fathers’ brain responses to their own 4- to 6-month-olds infants stimuli in social-cortical networks associated with mentalizing and empathy, including the insula, inferior-parietal lobule (IPL), dmPFC and IFG, suggesting that parents may share in real time their intuitive understanding of the infant’s state and signals (Atzil et al., 2012). This result may suggest that co-parenting evolved on the basis of the higher mammals’ capacity for a neural coordination with social partners and the ability to represent the other’s state in one’s physiology.

Studies assessing ERPs demonstrated some differences in the cortical response to infant stimuli between parents and non-parents (Proverbio et al., 2006, 2007; Grasso et al., 2009). In particular fathers and mothers showed larger amplitude 300 ms after the stimulus onset in parietal sites compared to non-parents when viewing unfamiliar infant faces (Proverbio et al., 2006, 2007). In a similar way, Grasso et al. (2009) enrolled 28 mothers (*M* = 36.61, *SD* = 8.26; 14 birth mothers and 14 foster/adoptive mothers) of children between the ages of 1.6 and 4.7 years (*M* = 2.7, *SD* = 0.9), and found in adoptive mothers larger P300 component when they were exposed to own compared to other infants faces, indicating a potential role of attachment at this level of processing (Weisman et al., 2012).

From these research emerges that adults, despite of their parental status or gender, are genetically and evolutionary programed to respond to human infants signals even considering some differences due to the experience.

## Parenthood Under Clinical Conditions

There are many psychopathological conditions that may affect the ability of parents to take care of their infants. There’s a wide range of literature investigating how psychopathological conditions can affect parental abilities from a behavioral perspective (Kowalenko et al., 2013) but in the last decade few studies has explored the neural basis of such impairing conditions (e.g., maternal depression and maternal drug addiction).

One of the most common and alarming diseases a mother could experiment in the perinatal period is the postpartum depression (PPD). It can have devastating and sometimes deadly consequences for mothers and babies when unidentified and untreated. PPD affects 13% of women worldwide within the first 12 weeks after giving birth, and 20% (which means one in five women) within the first postpartum year (Cole, 2015).

Many authors tried to investigate, in the last decade, which are the neural circuits interested by such condition in mothers responding to infants’ cues. For example, Moses-Kolko et al. (2010) examined 16 postpartum healthy mothers and fourteen unmedicated depressed mothers with fMRI blood-oxygen-level-dependent acquisition during a block-designed face versus shape matching task. A two-way analysis of variance was performed examining main effects of condition and group and group-by-condition interaction on activity in bilateral dorsomedial prefrontal cortical and amygdala regions of interest. All participants were medication-free, multiparous and breast- or bottle-feeding. Authors found that faces were associated with increased amygdala activity and shapes were associated with increased dorsomedial prefrontal activity in all women. But at the same time, they found that negative emotional faces activated the left dmPFC over a large region in Brodmann’s area 32, significantly less in depressed mothers than in healthy mothers (Moses-Kolko et al., 2010). They interpret this deficit in dmPFC activity as an index depressed mother’s present diminished awareness of empathic responses to other’s emotions (Donges et al., 2005). Again Laurent and Ablow (2013) studies these same sample’s responses to infant happy compared to distress facial expression; depressed mothers showed diminished responses to their own infants distress faces in the dorsal ACC compared to non-depressed mothers. Moreover, mothers with more severe symptomatology showed reduced responses to their own infant’s joy faces in the OFC and insula, circuits associated with motivation and self-regulation processes.

These authors conduced multiple studies comparing depressed and non-depressed mothers (Laurent and Ablow, 2012b, 2013); to examine the response to infant auditory stimuli, they compared 11 mothers with major depressive disorder to 11 mothers with no diagnosis recruited from the Women Infant Children (WIC) program, exposing them to their own 18-months-old infant’s cry, an unfamiliar infant’s cry and a control sound, during fMRI. Non-depressed mothers responded to their own infant’s cry grater than control sound in paralimbic areas (anterior insula and OFC), striatum, thalamus, midbrain [ventral tegmental area (VTA)], bilateral dmPFC, PCC and cerebellum. Compared to unfamiliar infant’s cry, these mothers showed greater responses to own infant’s cry in a more limited set of areas: ACC, right insula, right occipital fusiform and lingual gyrus and left posterior supramarginal gyrus. Depressed mothers failed, as a group, to show a significant response to own infant cry grater than both control sound and other infant’s cry. These results can be interpreted as whereas non-depressed mothers respond across multiple emotional responses and regulation circuits to infants crying while depressed mothers failed to respond to their infants. Depressed mothers’ failure to engage striatal and thalamic circuits in response to infants crying may underline motivational ad social bonding difficulties in mother–infant relationship (Laurent and Ablow, 2012b).

At the best of our knowledge, few studies have investigated the neural response of depressed mothers through the ERPs technique; Noll et al. (2012) investigated the role of parental status and depressive symptoms on early visual processing of infant faces in a sample of adult woman. They recruited from the New Haven community thirty adult women (17 mothers, 13 non-mothers, *M* age = 31.53 years, *SD* = 6.84). Participants underwent an EEG session and randomly selected infant face was presented for 1500 ms followed by another blank screen, which varied in duration between 500 and 700 ms. There were 75 trials in total each expressing one of three emotions: pleasure, comfort, or distress (25 exemplars for each expression). Authors observed a positive correlation between depressive symptoms severity and the N170 amplitude (Noll et al., 2012).

There’s also one other clinical issue affecting mother–infant relationship, with high rates of abuse, neglect, foster care placement and disturbed attachment toward the child; we’re talking about maternal drug addiction. Surprisingly, there is almost no literature about the neuroanatomical circuits affected by addiction in mothers. It is well know, though, that infant visual and auditory cues activate similar brain reward regions to drugs (e.g., cocaine), including the VTA, nucleus accumbens, cingulate and prefrontal cortices. Thus, in non-addicted mothers, exposure to infant cues appears to be reinforcing and important in activating healthy maternal reward and motivation circuits. In drug-addicted mothers healthy parent–infant interactions are disrupted by artificial stimulants of the dopaminergic system, such as cocaine, which may act as a highly reinforcing infant substitute (Swain et al., 2007).

Landi et al. (2011) investigated the degree to which neural circuits associated with parenting are disrupted in substance-using mothers. They included 26 substance-using (*M* age = 25.58 years, *SD* = 5.64) and twenty-eight non-using mothers (*M* age = 29 years, *SD* = 5.89) of children 1- to 3-months old. Substance use status was determined by a combination of self-report data and urine toxicology; women were considered substance-using if they used any teratogenic substance during pregnancy and/or into the post-partum period. Ten participants used only tobacco; four marijuana only; two alcohol only; one heroine, tobacco and cocaine; two heroine and tobacco; one tobacco and heroine; one amphetamine and tobacco; and five non-disclosed drugs. Using fMRI to examine the neural response to emotional infant cues (faces and cries), authors found that, in response to faces (of varying emotional valence) substance-using mothers, compared to non-using, showed reduced neural activation in prefrontal regions (dlPFC and vmPFC), visual processing (occipital lobes) and limbic regions (parahippocampus and amygdala). Similarly, in response to infant cries (of varying distress levels), the clinical group showed reduced activation in prefrontal regions auditory sensory processing regions, insula and limbic regions (parahippocampus and amygdala) (Landi et al., 2011). Such general reduced neural responsiveness may lead to difficulty in subsequent behavioral maternal response to the infant, and in the development of a healthy mother–infant attachment bonding.

Finally, Moser et al. (2013) examined, through fMRI, the influence of dissociation on neural activation of twenty mothers with post-traumatic stress disorder (PTSD) while viewing vide-stimuli of their children (aged 12–42 months) during stressful (separation) and non-stressful (play) mother–infant interactions. They found a positive association of limbic activation and PTSD symptom severity as well as negative association of limbic activation and dissociative symptom severity. Moreover, higher PTSD symptoms predicted activation in additional limbic areas, the enthorinal cortex, areas associated with emotional regulation and OFC. Activation in the enthorinal cortex originated from a positive correlation of neural activation with PTSD symptom severity during separation, while activation in the OFC originated from a negative correlation between PTSD symptom severity and play. Activation in the dlPFC originated from both a negative correlation of PTSD symptom severity with paly, and positive correlation of PTSD symptom severity with separation. While his diminished activation may be adaptive for the mother’s downregulation of physiological arousal due to her PTSD symptoms, it becomes maladaptive in the context of mother-child interaction (Moser et al., 2013).

It is not surprising there’s no literature investigating the diverse effects different types of drug abuse may have on the maternal abilities, it is maybe due to the wide variety of cerebral areas involved in this disease, the frequency of polydrug use which makes difficult to isolate the effect of one single substance and the peculiarity and fragility of such sample, all factors that make this research still to deepen.

## Conclusion

The main aim of this review was to present what is known about the neurobiology of mothering to neuroimaging techniques; we have delineated the complexity and abundance of the mechanisms underlying and influencing parental behavior and its connected physiological, psychological and behavioral adaptations.

From literature we reported, a set of brain circuits of parental response to baby stimuli, whether cries or faces, emerges: starting from the cingulate circuits involving midbrain and thalamus involved motivation and reward processes; frontal, insular, fusiform and occipital circuits engaged in social emotional/empathy responses and planning; memory processing regions including hippocampus, parahippocampus and amygdala implicated.

One of the first evidence which emerges from our exploration of the literature is that infant cues do not just orient the adult trough the infant, but also provides a wide range of information, including affective expression. Moreover, the overlapping neural circuitry for reward and affective processing provide an important link to understanding motivational factors underlying parental behavior. In addition to “liking” being a useful characterization of parental responsivity to infant cues, “wanting” also represents a hedonic dimension characterized by motivation to act. Infant visual and auditory stimuli can be used to selectively activate brain circuits related to arousal, mood, and social and habitual behaviors. Though, different groups we reported have used a mixture of stimuli including baby cries (of different distress level), laughter and images of different ages and different facial affect (happy vs. neutral vs. sad) and experience (own vs. familiar vs. unknown).

The nature of the adult–infant relation is complicated and relies on the integrity and function of physiological and behavioral systems in the domains of sensation, perception, affect, reward, executive function, motor output and learning. To engage in parenting behaviors, adults have to be sensitive to infant cues and emotionally prepared and motivated to engage socially with the infant; adults must selectively attend to the infant in the context of competing stimuli, and finally, they must be restrained and consistent in their responsiveness. Consequently, when a mother is at risk to engage in dysfunctional parenting, such as when she is depressed or has a history of drug addiction, the function of many or all of maternal and related systems may be compromised. In fact, the studies we reported showed noticeable differences in the neural responses to infant stimuli in mothers who presented a history of psychopathology in those areas mentioned above, involved in parental understanding and reaction.

Further research is needed to deeply explain the complex “panorama” underlying the neural mechanisms regulating the biological response of parents to their infants’ signals, needs and requests. One of the possible future perspectives we are approaching is the possibility to investigate neural activity of mothers in ecological situations. For example, by using an innovative and specific EEG cap endorsed in the “MOVE” system, it will be possible to monitor neural parent–infant dyadic activity during interactive, face-to-face and every-day sessions and this would be essential to more deeply understand which mechanisms take place during real dyadic exchanges.

## Conflict of Interest Statement

The authors declare that the research was conducted in the absence of any commercial or financial relationships that could be construed as a potential conflict of interest.
